# Percutaneous ablation of intrathyroidal parathyroid adenoma: A case report and brief review of the literature

**DOI:** 10.3892/mi.2025.285

**Published:** 2025-11-11

**Authors:** Aras J. Qaradakhy, Abdulwahid M. Salih, Shaho F. Ahmed, Rebaz M. Ali, Ari M. Abdullah, Aso N. Qadir, Imad J. Habibullah, Hawkar A. Nasralla, Abdullah A. Qadir, Fahmi H. Kakamad

**Affiliations:** 1 Department of Scientific Affairs, Smart Health Tower, Sulaymaniyah 46001, Iraq; 2 Department of Radiology, Shorsh Teaching Hospital, Sulaymaniyah 46001, Iraq; 3 Department of Pathology, Sulaymaniyah Teaching Hospital, Sulaymaniyah 46001, Iraq; 4 College of Medicine, University of Sulaimani, Sulaymaniyah 46001, Iraq; 5 Kscien Organization for Scientific Research (Middle East Office), Sulaymaniyah 46001, Iraq

**Keywords:** primary hyperparathyroidism, parathyroid adenoma, microwave ablation, intrathyroidal adenoma, hypercalcemia, minimally invasive therapy

## Abstract

Primary hyperparathyroidism (HPT), predominantly caused by parathyroid adenomas (PAs), leads to elevated calcium levels and a range of complications, including renal, skeletal and cardiovascular issues. While parathyroidectomy is the standard treatment for resolving hypercalcemia, the present study describes a case of intrathyroidal PA treated with microwave ablation (MWA). A 29-year-old woman with recurrent HPT presented with elevated parathyroid hormone (183 pg/ml) and serum calcium (11.06 mg/dl) levels following a previous right upper and lower parathyroidectomy. Imaging revealed a well-defined hypoechoic nodule within the right thyroid lobe, confirmed as an intrathyroidal PA following sestamibi scanning. The patient underwent MWA, achieving the rapid normalization of parathyroid hormone and calcium levels. Although PA is a common cause of primary HPT, a thorough review of the literature found no instances of intrathyroidal PA treated with MWA. On the whole, the present case report demonstrates that MWA may be a safe and effective alternative for the treatment of intrathyroidal PAs, particularly when traditional surgery is not a viable option.

## Introduction

Parathyroid adenomas (PAs) are the most common cause of primary hyperparathyroidism (HPT), an endocrine disorder that frequently leads to hypercalcemia of varying severity based on the progression of the condition (1). Complications affecting the heart, kidneys, and bones, as well as a tendency for peptic ulcer disease, are frequently linked to primary HPT. In rare instances, hypercalcemia can escalate into a hypercalcemic crisis, a critical emergency requiring immediate medical attention (2).

Ectopic parathyroid glands occur from abnormal migration during early development, with an incidence of ~2 to 43%. Ectopic inferior parathyroid glands are most commonly located in the anterior mediastinum, often associated with the thymus or thyroid gland. By contrast, ectopic superior parathyroid glands are typically found in the tracheoesophageal groove or the retroesophageal region (3). The PAs can be located posterior to the thyroid lobes or in ectopic locations. Intrathyroidal PAs are relatively rare, accounting for 1 to 6% of all PA cases. Despite their rare occurrence, they can present diagnostic challenges that complicate management (4).

In patients with thyroid nodules and abnormal parathyroid-related laboratory findings, the suspicion of an intrathyroidal parathyroid may arise (5). Parathyroid imaging techniques have advanced, providing for more precise localization for surgery. In the case of intrathyroidal PAs, ultrasound (US) is the preferred imaging modality (6).

The standard treatment for primary HPT is the surgical removal of the PA (7). In recent years, advancements in thermal ablation technology have led to the use of microwave ablation (MWA) and other techniques, such as radiofrequency ablation (RFA) and laser ablation, in treating HPT. These methods have proven to be safe and effective for managing primary HPT in patients who are either ineligible for surgery or who do not opt to undergo surgical intervention (8).

The present study describes a case of intrathyroidal PA successfully treated with US-guided MWA. The eligibility of all references has been evaluated, and the report is structured following the CaReL guidelines (9,10).

## Case report

### Patient information

A 29-year-old woman presented to Smart Health Tower (Sulaymaniyah, Iraq), in September, 2024 with recurrent HPT and bilateral loin pain. An analysis of her medical history revealed a prior diagnosis of HPT, while her surgical history included a right upper and lower parathyroidectomy, tonsillectomy, cesarean section and two anterior vaginal wall repairs.

### Clinical findings

The vital signs of the patient were stable, and a neck examination did not reveal any notable findings. Physical examinations of the chest, abdomen, skin and extremities did not reveal any abnormalities.

### Diagnostic approach

**Figure 1:**
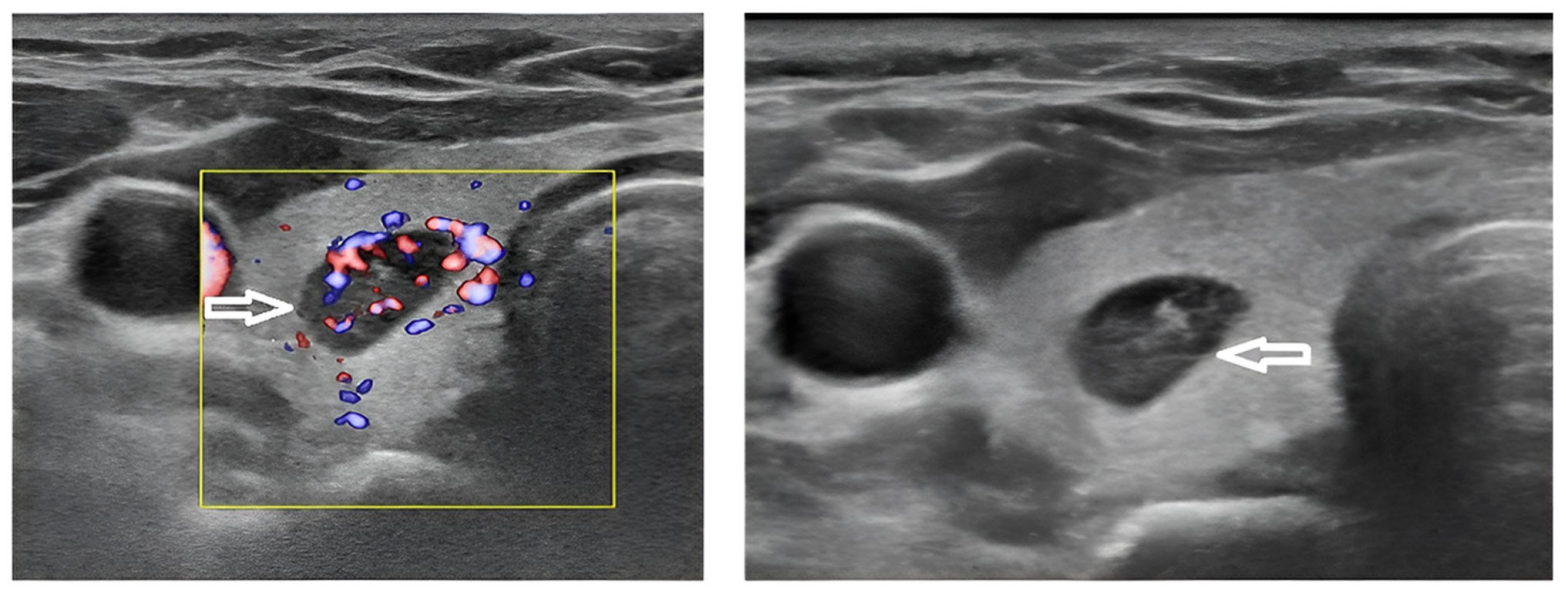
Ultrasound images illustrating a small, well-defined, solid hypoechoic nodule measuring 10x7.7x4.8 mm with a regular surface and prominent hypervascularity, located in the mid-third of the right thyroid lobe. The image on the left panel (color Doppler mode) illustrates the nodule indicated by the white arrow, while the yellow boxed area marks the region of interest used for Doppler evaluation, demonstrating multiple intranodular color flow signals consistent with marked hypervascularity. The image on the right panel (grayscale mode) depicts the same hypoechoic nodule (white arrow) with smooth, well-defined margins and homogeneous echotexture, without calcification or cystic changes. These imaging characteristics are compatible with an intrathyroidal parathyroid adenoma.

**Figure 2:**
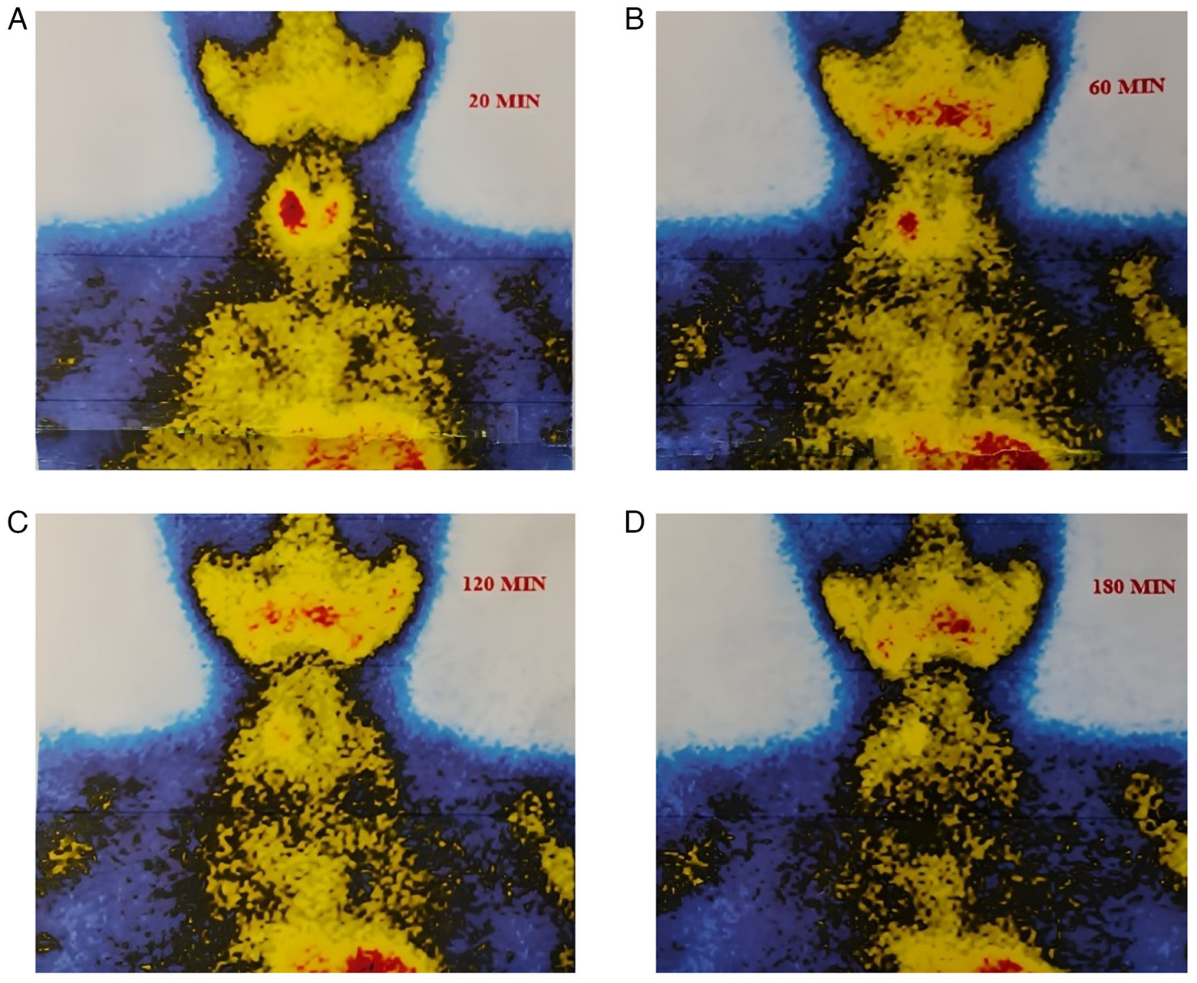
Sequential 99mTc-sestamibi scintigraphy images obtained at different time intervals demonstrating radiotracer uptake and washout patterns in the thyroid region. (A) At 20 min (early phase), there is intense radiotracer uptake in both thyroid lobes, with a focally increased accumulation in the right thyroid lobe, suggesting a hyperfunctioning lesion. (B) At 60 min, partial washout of tracer activity is observed from most of the thyroid tissue, while the right thyroid lobe shows persistent uptake. (C) At 120 min, further washout from normal thyroid tissue occurs, with sustained focal uptake in the right lobe. (D) At 180 min, complete washout from the thyroid gland is seen, except for persistent radiotracer retention in the mid-portion of the right lobe, confirming the presence of an intrathyroidal parathyroid adenoma.

The parathyroid hormone (PTH) level of the patient was elevated at 183 pg/ml (reference range, 15-65 pg/ml), with a serum calcium concentration of 11.06 mg/dl (reference range, 8.5-10.5 mg/dl) and the thyroglobulin level measured at 2.62 ng/ml (reference range, 3.5-77 ng/ml). A neck US revealed that both thyroid lobes were of normal size with a homogeneous echotexture, along with a single well-defined solid hypoechoic nodule in the right mid-third of the thyroid, measuring 10x7.7x4.8 mm. The nodule, which was hypervascular on color Doppler, was suspected to be an intrathyroidal parathyroid nodule (TR4) (Fig. 1). This nodule had previously been diagnosed as a benign follicular nodule with thyroiditis on fine needle aspiration (FNA). A sestamibi scan demonstrated increased radiotracer accumulation in the right thyroid lobe on the initial image, with washout observed from the thyroid gland, apart from the right lobe, on the delayed image (Fig. 2).

### Therapeutic interventions

Due to the previous parathyroidectomy, repeat surgery was considered technically challenging and associated with a higher risk of complications, including recurrent laryngeal nerve injury, post-operative hypocalcemia, scarring-related difficulties and potential cosmetic concerns. The patient, therefore, preferred a minimally invasive approach under local anesthesia to avoid another neck surgery.

Using US guidance and local anesthesia, a Microwave Therapeutic System ECO-200G (ECO Medical Instruments Co., Ltd.) equipped with an 18-gauge internally cooled needle antenna was employed to ablate the solid hypoechoic intrathyroidal nodule on the right side. The antenna was advanced through a trans-isthmic puncture path to ensure stability and maintain a safe distance from critical structures, including the recurrent laryngeal nerve and trachea.

The microwave energy output was set at 25 W, with a total ablation time of 10 sec. The ablation endpoint was determined by real-time US visualization of a hyperechoic zone (‘cloud sign’) surrounding the antenna tip, indicating adequate coagulation of the target tissue. Continuous US monitoring was maintained throughout the procedure to confirm complete ablation and to prevent excessive thermal spread.

**Figure 3:**
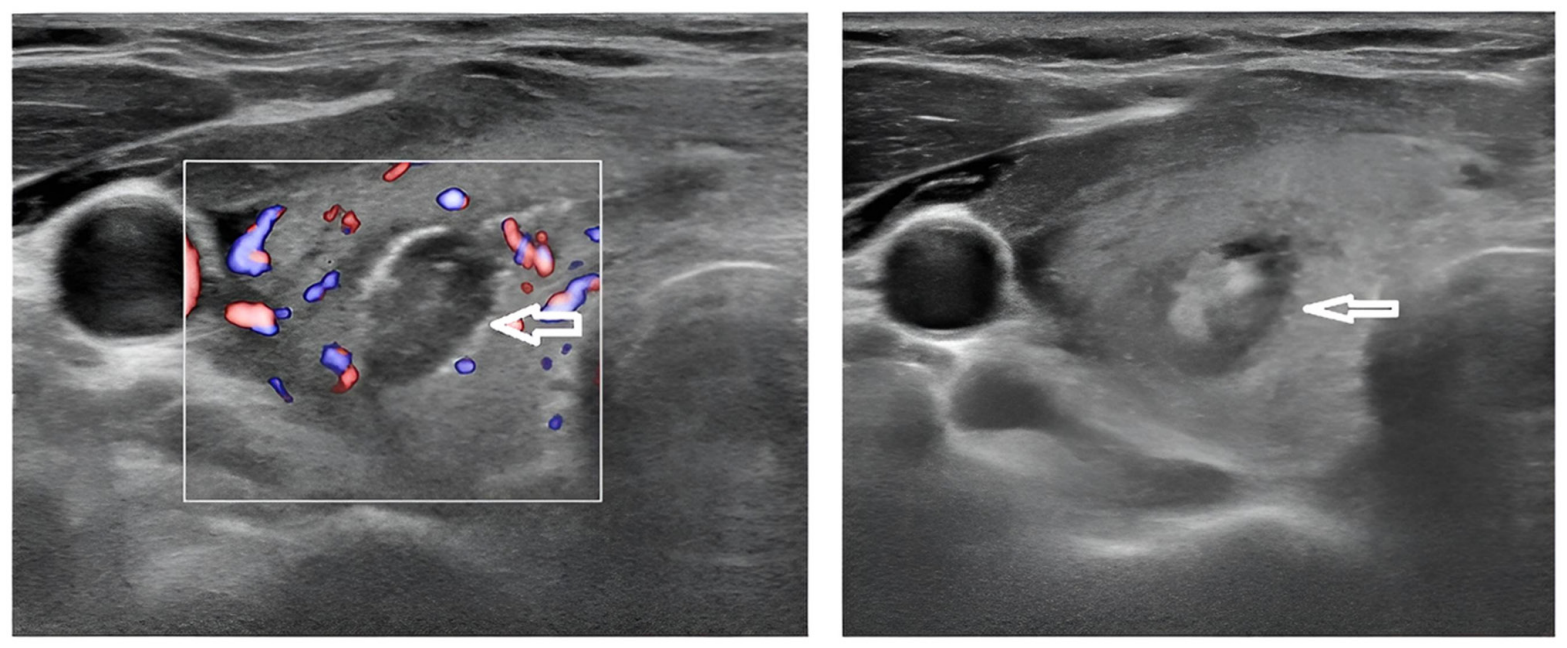
Ultrasound images of the right thyroid lobe obtained immediately following microwave ablation, illustrating post-procedural changes in the treated nodule. The image on the left panel (color Doppler mode) illustrates the ablated nodule indicated by the white arrow, demonstrating marked reduction in internal vascularity compared with the pre-ablation study. The boxed white area represents the region of interest used for Doppler assessment, confirming diminished or absent blood flow within the ablated zone. The image on the right panel (grayscale mode) depicts the same nodule (white arrow) with inhomogeneous echotexture and a hyperechoic halo corresponding to coagulative necrosis, consistent with a successful ablation outcome.

Immediate post-ablation US demonstrated inhomogeneous echotexture and reduced vascularity, confirming successful ablation (Fig. 3). As a result, the serum PTH level of the patient decreased to normal levels (4 pg/ml) within a few hours, and the serum calcium level normalized (9.88 mg/dl) during follow-up.

### Follow-up and outcome

Following MWA, the patient recovered uneventfully with no reported complications. At 3 weeks following the procedure, both the serum calcium (9.35 mg/dl) and PTH levels (30.5 pg/ml) remained within normal ranges. The patient is currently under active follow-up. Serial measurements of serum PTH and calcium levels were scheduled at 3-, 6- and 12-month post-ablation to monitor for biochemical recurrence.

## Discussion

Primary HPT predominantly affects women and older adults, with a prevalence of ~1% in individuals aged ≥69 years (11). The case presented herein was a 29-year-old female patient with a history of HPT and prior right upper and lower parathyroidectomy.

The complications associated with HPT include the formation of renal stones, reduced bone density leading to osteoporosis and persistent bone pain. Additionally, this condition may contribute to the development of hypertension, increasing the risk of cardiovascular issues. These complications arise due to prolonged elevated calcium levels and excessive secretion of PTH, which can significantly affect both the renal and skeletal systems, as well as overall cardiovascular health (12).

Biochemical testing is a cornerstone in the diagnosis of primary HPT and assessing the functionality of PAs. Serum calcium is the most direct indicator of parathyroid dysfunction, with elevated levels suggesting HPT. In cases of suspected PAs, high serum calcium levels serve as a prompt for further diagnostic investigation. Elevated calcium levels, particularly when persistent, indicate an imbalance due to autonomous PTH secretion. The excess calcium in the blood can lead to severe symptoms and complications affecting the renal, skeletal and cardiovascular systems (4). The patient in the present case report exhibited elevated PTH and serum calcium levels without any complications affecting the renal, skeletal or cardiovascular systems.

Several imaging techniques are essential for improving pre-operative localization, including US, sestamibi scanning and contrast-enhanced computed tomography scan. However, due to overlapping imaging characteristics with thyroid lesions, intrathyroidal PAs may still evade accurate detection, posing a diagnostic challenge (11). In the US, intrathyroidal PAs can resemble other abnormalities, such as benign thyroid nodules, which are often found concurrently with intrathyroidal PAs. Indicators suggestive of an intrathyroidal PA include a solid lesion lacking cystic components, hypoechogenicity, and the identification of a single polar feeding artery on Doppler imaging (13). In the present case report, a neck US revealed a homogeneous echotexture and a single, well-defined solid hypoechoic nodule in the right mid-third of the thyroid, measuring 10x7.7x4.8 mm. The nodule appeared hypervascular on color Doppler. However, the sestamibi scan revealed increased radiotracer uptake in the right thyroid lobe on the initial scan, with a washout from the thyroid gland observed on the delayed image, apart from the right lobe.

FNA of the lesion can provide insight into the cell type, potentially supporting the diagnosis. However, distinguishing between parathyroid and thyroid tissue is challenging due to marked overlap in cytological and architectural features. Characteristics once considered indicative of thyroid tissue, such as colloid, follicles and perivacuolar granulation, are frequently observed in parathyroid samples. Additionally, a number of PAs exhibit similarities to follicular, papillary and medullary thyroid carcinomas (13). In the present case report, FNA had previously diagnosed the nodule as a benign follicular nodule with thyroiditis.

The standard treatment for primary HPT is the surgical removal of one or more PAs. It is estimated that skilled surgeons successfully identify the affected gland in 95% of cases. However, the morbidity and mortality rates associated with parathyroid surgery are higher in elderly patients (14). In recent years, local anesthesia and minimally invasive nonsurgical therapies have been increasingly utilized to treat primary HPT. However, the effectiveness of these minimally invasive non-surgical approaches remains a subject of debate (14). The patient in the present case report had a recurrent intrathyroidal PA following a previous parathyroidectomy, rendering repeat surgery technically challenging and associated with an increased risk of complications, such as recurrent laryngeal nerve injury, hypocalcemia and scarring-related difficulties.

US-guided laser ablation can temporarily lower serum PTH and calcium levels; however, it does not provide a permanent resolution of HPT. Consequently, laser ablation cannot be considered a definitive treatment for primary HPT (14). Other non-surgical treatments, including RFA and high-intensity focused US, have been previously introduced. However, clinical experience with these methods remains limited, as only a small number of patients have undergone these treatments (14).

MWA has emerged as a promising, minimally invasive alternative for cases where traditional surgery has not succeeded. Unlike RFA, which has been explored more extensively in endocrine pathology, MWA enables greater tissue penetration and more rapid heating times, reducing the duration of treatment and the risk of damage to adjacent structures (6,15). The therapeutic effect of MWA on PAs is principally mechanical and thermal. MWA produces dielectric heating via the oscillation of water molecules, which raises intralesional temperatures rapidly and leads to coagulative necrosis and protein denaturation in the central treatment zone. This direct thermal injury destroys parathyroid chief cells and thereby abolishes autonomous PTH secretion (16).

In comparing MWA with traditional surgery and other ablation modalities for intrathyroidal PA, several recent studies have provided helpful insight. A large retrospective cohort study on 212 patients with primary HPT compared MWA with parathyroidectomy, with a median follow-up of 28.5 months (17). Following propensity-score matching, there was no statistically significant difference in clinical cure rates between the two groups; persistent or recurrent disease rates were also comparable (17). This suggests that MWA may provide long-term efficacy similar to surgery, at least in well-selected patients.

In another prospective multicenter study including 132 patients with primary HPT, the efficacy and safety of MWA and RFA were compared over follow-up periods ranging from 6 to 36 months (median, ~12 months). The overall cure rate was ~80%, with no significant differences observed between the MWA and RFA groups in terms of cure or complication rates. Notably, the pre-ablation PTH level was identified as the primary prognostic factor (18).

In their study, Liu *et al* (7) treated 15 patients with benign parathyroid nodules, concluding that MWA is a safe and effective technique for managing primary HPT associated with parathyroid nodules. The procedure was shown to reduce adenoma size, lower serum PTH and calcium levels, and alleviate symptoms related to the nodules (7). However, in another study, Yu *et al* reported that MWA may be a safe and effective option for managing recurrent and persistent nodules associated with secondary HPT (14). In the present case report, MWA was used to treat intrathyroidal PA, leading to a reduction and normalization of both PTH and serum calcium levels. Furthermore, a review of the literature revealed no reported cases of intrathyroidal PAs treated with MWA. However, 6 reported cases of intrathyroidal PAs were included in the literature review (Table I) (2,4-6,19).

MWA for PAs and HPT generally demonstrates a favorable safety profile; however, evidence indicates that certain risks require careful management. In a previous systematic review of US-guided MWA for secondary HPT involving 26 studies and 932 patients, hypocalcemia occurred in 35.2% of cases and transient hoarseness in ~9.2% of cases, with no major complications or mortality reported (20). Similarly, a previous retrospective study including 264 patients with primary and secondary HPT reported an overall complication rate of 12.1%, with major complications comprising aphonia or hoarseness in some patients and severe hypocalcemia in 18.2%; one instance of permanent recurrent laryngeal nerve injury was documented (21). In the present case report, no complications such as hoarseness or clinically significant hypocalcemia were observed.

The present case report has several limitations. First, the short follow-up duration restricted the assessment of long-term biochemical and structural outcomes following MWA. Second, as a single-patient observation, the present case report lacks statistical validity and cannot yield generalizable conclusions regarding the efficacy or safety of MWA in treating intrathyroidal PA. Furthermore, imaging interpretation was based primarily on US and sestamibi scan findings, which, while standard, may have inherent limitations in sensitivity and specificity for intrathyroidal lesions. Additionally, the lack of standardized anterior-posterior and oblique views in the presented sestamibi scan, as well as the absence of a uniform color scale to indicate the intensity of radioactive distribution, may limit the visual standardization and comparability of the imaging data.

In conclusion, MWA may be a safe and effective alternative for the treatment of intrathyroidal PAs, particularly when traditional surgery is not a viable option.

## Figures and Tables

**Table I:** Summary of 6 cases of intrathyroidal parathyroid adenoma reported in the literature.

First author/year of publication	Country	Age, years	Sex	Clinical presentation	S. Ca (reference range)	PTH (reference range)	Neck U/S	Management	Outcome	(Refs.)
Papanikos, 2024	Greece	67	Male	Palpable right neck mass	14.4 mg/dl (8.5-10.5 mg/dl)	451 pg/ml (15-65 pg/ml)	Multilobulated cystic mass 6.9x3.0x2.6 cm, lower right thyroid lobe	Partial parathy-roidectomy + total thyroidectomy	Normal Ca and PTH after surgery	(2)
Papanikolaou, 2020	Greece	43	Female	Right thyroid-lobe mass	12.97 mg/dl (8.5-10.5 mg/dl)	457.2 pg/ml (15-65 pg/ml)	Predominantly cystic nodule 3.7x2.5x4 cm with increased peripheral vascularity	Thyroidectomy + central neck dissection	Normal Ca and PTH after surgery	(4)
Kim, 2024	Korea	60	Male	A thyroid nodule	10.4 mg/dl (8.5-10.5 mg/dl)	1,172 pg/ml (15-65 pg/ml)	Right mid-lower-pole 2.6 cm + left upper-pole 1.4 cm nodules	Right hemithy roidectomy + multi-gland resection	Normal Ca and PTH after surgery	(5)
Eldeiry, 2024	USA	64	Female	Long-standing primary HPT	N/A	85 pg/ml (15-65 pg/ml)	6-mm vascular nodule, left thyroid lobe	RFA	Normal Ca and PTH after RFA	(6)
Shi, 2016	China	59	Female	Incidental right thyroid nodule	Not detected	Not detected	Hypoechoic nodule 1.4x0.9 cm, right lobe	Surgical excision	N/A	(19)
Shi, 2016	China	45	Female	Twitching, palpable left neck mass	10.68 mg/dl (8.5-10.5 mg/dl)	182.8 pg/ml (15-65 pg/ml)	Hypervascular mass 1.8x1.1 cm, left lobe	Surgical excision	Normal Ca and PTH after surgery	(19)

U/S, ultrasound; S. Ca, serum calcium; Ca, calcium; PTH, parathyroid hormone; N/A, not available; RFA, radiofrequency ablation; HPT, hyperparathyroidism.

## Data Availability

The data generated in the present study may be requested from the corresponding author.
